# Perceptions of an AI-based clinical decision support tool for prescribing in multiple long-term conditions: a qualitative study of general practice clinicians in England

**DOI:** 10.1136/bmjopen-2025-102833

**Published:** 2025-11-23

**Authors:** Alexander d’Elia, Simon George Morris, Jennifer Cooper, Krishnarajah Nirantharakumar, Thomas Jackson, Tom Marshall, Leah Fitzsimmons, Louise J Jackson, Francesca Crowe, Shamil Haroon, Sheila Greenfield, Ellie Hathaway

**Affiliations:** 1 College of Medicine and Health, University of Birmingham Institute of Applied Health Research, Birmingham, UK; 2 University of Birmingham Institute of Applied Health Research, Birmingham, England, UK; 3 University of Birmingham Institute of Inflammation and Ageing, Birmingham, England, UK; 4 University of Birmingham Unit of Public Health Epidemiology and Biostatistics, Birmingham, England, UK; 5 University of Birmingham Health Economics Unit, Birmingham, England, UK

**Keywords:** Primary Health Care, Artificial Intelligence, QUALITATIVE RESEARCH, Multimorbidity

## Abstract

**Abstract:**

**Background:**

Artificial intelligence (AI)-based clinical decision support systems (CDSSs) are currently being developed to aid prescribing in primary care. There is a lack of research on how these systems will be perceived and used by healthcare professionals and subsequently on how to optimise the implementation process of AI-based CDSSs (AICDSSs).

**Objectives:**

To explore healthcare professionals’ perspectives on the use of an AICDSS for prescribing in co-existing multiple long-term conditions (MLTC), and the relevance to shared decision making (SDM).

**Design:**

Qualitative study using template analysis of semistructured interviews, based on a case vignette and a mock-up of an AICDSS.

**Setting:**

Healthcare professionals prescribing for patients working in the English National Health Service (NHS) primary care in the West Midlands region.

**Participants:**

A purposive sample of general practitioners/resident doctors (10), nurse prescribers (3) and prescribing pharmacists (2) working in the English NHS primary care.

**Results:**

The proposed tool generated interest among the participants. Findings included the perception of the tool as user friendly and as a valuable complement to existing clinical guidelines, particularly in a patient population with multiple long-term conditions and polypharmacy, where existing guidelines may be inadequate. Concerns were raised about integration into existing clinical documentation systems, medicolegal aspects, how to interpret findings that were inconsistent with clinical guidelines, and the impact on patient-prescriber relationships. Views differed on whether the tool would aid SDM.

**Conclusion:**

AICDSSs such as the OPTIMAL tool hold potential for optimising pharmaceutical treatment in patients with MLTC. However, specific issues related to the tool need to be addressed and careful implementation into the existing clinical practice is necessary to realise the potential benefits.

**STRENGTHS AND LIMITATIONS OF THIS STUDY**

- This study explored perspectives of both medical and non-medical prescribers in relation to use of an artificial intelligence-informed clinical decision support system.
- The presentation of a system interface supported exploration of more practical issues of usability.
- Participants were drawn from a single UK region and purposively sampled on professional background only.
- This study is unable to reliably distinguish any potential differences between professional groups due to relatively small sample.

## Background

 The co-occurrence of two or more concurrent long-term health conditions in an individual is increasingly common and presents a growing challenge for patients, clinicians and for health services.^1^ With multiple long-term conditions (MLTC) comes the prescription of multiple medications (polypharmacy)^2^ and subsequently increased risk of drug interactions and adverse outcomes.^3^ Pharmaceutical decision-making is a particular challenge for clinicians managing MLTC, with a growing gap between clinical guidelines for single conditions and the clinical complexity of the patient in the clinic.^3^,^5^

Clinical decision support systems (CDSS), developed to augment the complex decision-making processes undertaken by clinicians,^6^ represent an opportunity to support clinicians. A systematic review examining the adoption of CDSS in the context of MLTC found a lack of evaluations of usability (as well as effectiveness) and highlighted the need for more evidence to support their development.^7^ Decision aids for patients are a related concept, aimed at providing tailored, evidence-based information to patients on different therapeutic options.^8^ A systematic review of their use in primary care settings concluded they are effective in improving knowledge of the disease and treatment options, and awareness of risk, potentially increasing patient agency and aiding shared decision making (SDM).^9^ However, qualitative studies exploring healthcare professionals’ perceptions of CDSS have highlighted concerns with usability, integration and maturity of algorithms and systems as a limiting factor for uptake. A meta-synthesis of these studies highlighted the need for further research on problem solving in real-world clinical settings and to understand how CDSS could be presented to patients and clinicians in user interfaces.^10^

Artificial intelligence (AI) methods are now being applied to the study of MLTC, to increase understanding of disease clusters, predict their trajectories and outcomes, and to optimise therapies.^11^ OPTIMAL (optimising therapies, disease trajectories and AI-assisted clinical management for patients living with complex multimorbidity) is a research project with one aim to develop a CDSS to aid pharmaceutical clinical decision-making in patients with MLTC (also termed multimorbidity). This prediction model and attached systems are hereafter referred to as ‘the OPTIMAL tool’ or ‘the tool’. The OPTIMAL tool is being developed using machine learning methods with medical and prescription data from CPRD Aurum (Clinical Practice Research Datalink Aurum: primary care records from practices in England) to provide risk predictions of pharmaceutical treatments for a given patient where there is more than one treatment option. The aim of the tool is to produce individual predictions of different pharmaceutical therapies’ effect on the primary morbidity for the consultation, existing co-morbidities and the risk of developing further comorbidity, all of which is presented to the clinician through a graphical user interface.^11^

This study builds on previous research by the OPTIMAL team exploring clinicians’ perceptions of managing MLTC and their attitudes towards using AI tools to support shared decision making and parallel research with primary care patients to explore their perceptions of artificial intelligence-based clinical decision support system (AICDSS) and specifically their perspectives of the OPTIMAL tool.^12^

## Aim

The aim of this study was to assess healthcare professionals’ perceptions of the usability of the OPTIMAL AI tool (as proposed in a mock-up version), with a particular focus on data interpretation, practical implementation and the tool’s impact on SDM in the consultation. A secondary aim was to uncover wider and more generalisable implementation aspects of AICDSSs.

## Methods

### Study design

This is a qualitative study drawing on individual, semistructured interviews. The study was underpinned by a phenomenological approach,^13^ emphasising an intention to seek to understand the experiences and perspectives of participants in relation to the tool and the wider context of shared prescribing decisions for patients with MLTC.

### Participants and recruitment

A total of 15 prescribing primary care clinicians working in the National Health Service in the West Midlands region of England participated in the study. General practitioners represented the largest group, with 10 participants of which two were resident doctors completing general practitioner (GP) training programmes. Non-GP prescribers were represented by three primary care nurse practitioners and two primary care pharmacists (hereafter ‘nurse and pharmacy prescribers’, NPPs). Six interviewees were male and nine were female, and the most common ethnicities were Asian and British, both with six participants each. The most common age group was 25–34 years (n=6), followed by 45–44 years (n=3). Most (n=9) participants had more than 10 years of clinical experience. See table 1 for summary of demographics.

**Table 1:** Demographics of participants

Characteristic	Subgroup	Frequency n (%)
Total		15
Gender	Male	6 (40)
	Female	9 (60)
Professional role	General practitioner (GP) GP registrar	7 (47) 3 (20)
	Nurse practitioner	3 (20)
	Prescribing pharmacist	2 (13)
Ethnicity	Asian, Asian British, Asian Welsh	6 (40)
	Black, Black British, Black Welsh, Caribbean or African	0 (0)
	Mixed or multiple	0 (0)
	White	6 (40)
	Other ethnic group	1 (7)
	Did not answer	2 (13)
Age group	25–34	6 (40)
	35–44	2 (13)
	45–54	3 (20)
	55+	2 (13)
	Did not answer	2 (13)
Time since completion of training (eg, finishing medical school)	2–5 years	2 (13)
	5–10 years	4 (27)
	>10 years	9 (60)

Sampling was purposive, in that we set out to recruit 10 GPs and five NPPs working in primary care settings. The mix of professional roles was intended to reflect that the majority of prescribing is undertaken by general practitioners, while also seeking to gather views of other professional prescriber groups. Demographic information was recorded to better characterise the sample in terms of its diversity and representativeness, but the recruitment was not itself guided by it. In establishing the sample size, we were guided by considerations of information power.^14^ In this study, the sample specificity is relatively dense in that participants are all working in similar primary care settings in the context of MLTC management, and in that the study was focused on a single example of an AICDSS and single example patient-case vignette.

This study followed a previous round of semistructured interviews with healthcare professionals undertaken as part of the OPTIMAL study which explored general views on AI and healthcare.^15^

Participants in this first stage were recruited by requests sent to national healthcare organisations to advertise the study to members, and through local healthcare professional networks in the West Midlands. 13 participants in the current study had previously participated in the aforementioned interview study, but two participants recruited via local networks had not. This reflects an element of convenience sampling.^16^

The study information sheet and consent form (online supplemental 1) were sent in advance. Consent to participate was confirmed verbally prior to the start of the interview and written consent forms signed and returned electronically by participants. A £15 shopping voucher was provided to participants in recognition of their time.

### Data collection

15 individual semistructured interviews with a duration of 30–44 min (mean 35) using a topic guide were conducted with participants between February and June 2024. The option of face-to-face or online interviews was given and all but one participant opted for online. Online interviews were conducted using Zoom^17^ and recorded, with the audio sent for transcription to a contracted external provider.

The interview topic guide (online supplemental 2) was developed in collaboration with the Patient Advisory Group (PAG) for the OPTIMAL study (reference) and the wider OPTIMAL team. The PAG consisted of nine members who were either living with MLTCs (four or more long-term conditions) or caring for people with MLTCs, with experience of common as well as rare and both physical and mental conditions, and with variation in personal characteristics (age, sex, ethnicity). It provided indicative questions and prompts covering perspectives on shared decision making, the use of non-AI clinical decision support tools, and perspectives on the proposed OPTIMAL tool. The topic guide was piloted on three GPs which led to minor semantic adjustments.

**Figure 1:**
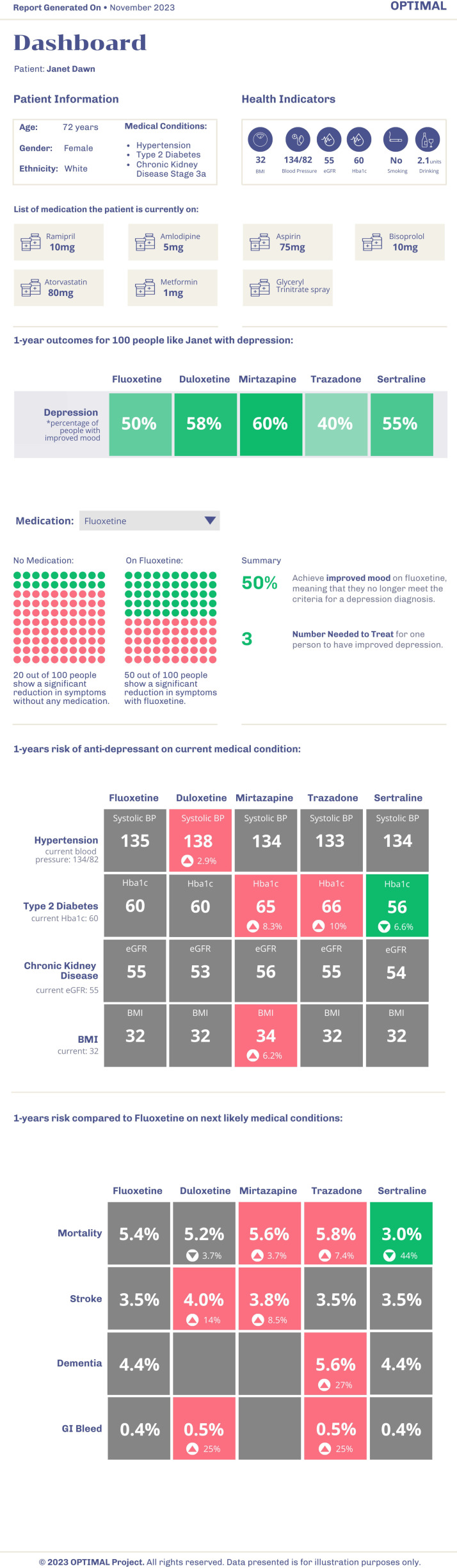
The mock-up of the OPTIMAL (OPTIMising therapies, disease trajectories, and AI assisted clinical management for patients Living with complex multimorbidity tool as used in the interviews, together with the fictional patient vignette. From top to bottom: demographics and key health indicators, current medication, predicted effect on main condition of current consultation (depression), number-needed-to-treat, predicted effect on key health indicators of different pharmaceutical options, and predicted risk of ‘next likely medical condition’. Form factor chosen to fit tool in a vertically oriented window on the clinician’s computer screen, next to existing electronic health record.

After an introduction covering familiarity with using non-AICDSSs, an outline of the OPTIMAL tool and general views on SDM, a clinical case vignette based on a patient with MLTC (diabetes type 2, obesity, ischaemic heart disease and hypertension) presenting with moderate-severe depression was presented to the participant. It reflected a common dilemma for GPs in making a decision outside of usual guidelines for prescribing antidepressants. Vignettes have previously been used in evaluating clinical applications of AI in MLTC.^18^ In parallel, a mock-up of the proposed clinical decision support tool (figure 1) was shared onscreen with participants. It included summary medical and prescribing information, measures of the effectiveness of comparator treatments in a group of patients with similar clinical characteristics, and estimated effects on current long-term conditions, and the most likely next conditions. Our vignette was created through an iterative process through discussions between the practising clinicians (including 4 GPs) in the OPTIMAL team and the PAG. The mock-up was then developed through iterative discussions between the clinicians, and a software engineer and the PAG, based on a literature review of existing shared decision-making tools including QRISK. Several ways of representing data through visual means were trialled and iterated, and those that most effectively conveyed the outputs and were accessible to the clinicians and the PAG were used. This was done as part of the wider OPTIMAL project.^15^

As per the interview guide, the participants were then asked questions on perceived usability of the tool, including implementation factors, role in SDM, general appearance and wider aspects of using AICDSSs in clinical practice.

Both interviewers were male and had experience of conducting and analysing qualitative interviews as part of PhD (AdE) and MSc (SGM) study and were on National Health Service medical training pathways at the time of data collection, with AdE working in general practice and SGM in public health (and previously psychiatry). 10 interviews were completed by AdE and five by SGM.

### Data analysis

After the interviews had been conducted, the data were coded by the researcher who had conducted them. NVivo 14 software^19^ was used to manage the data, with verbatim transcripts imported. Data were analysed through template analysis, a form of codebook-led thematic analysis as described by King and Brooks (2018).^20^ Template analysis was chosen as it allowed first the development of codes to meet the primary aim of the study to be deductively applied to the data, while subsequently allowing for inductive flexibility in exploring emerging themes in the data (in particular supporting the secondary aim as stated in the Aims section above).

An initial coding template with themes was created based on the topic guide and our initial familiarisation with the data. The themes were organised into two overarching groups reflecting the aims: (1) views on the OPTIMAL tool and its practical implementation in clinical practice and (2) the interpretation and application of the outputs from an AICDSS in general such as the OPTIMAL tool.

Two interviews were coded by both researchers (AD and GM) and reviewed for consistency. Codes were compared through consensus meetings as similar codes emerged. Throughout the coding process, themes were then modified to reflect the codes derived from the data, and subthemes were created to describe the findings in detail.

The results of the analysis were presented to the PAG which provided feedback supporting further refinement of the themes. No member checking was undertaken with participants but the option to receive the study report was given.

The study did not aim to compare the views of different professional groups, but overarching differences that were observed were reported in the results.

### Patient and public involvement

The OPTIMAL study has a Patient Advisory Group who have contributed to the design and development of this study, including aiding the development of the topic guides, and provided feedback on the initial findings of this study.

## Results

The template analysis resulted in a table with four different overarching themes reflecting the primary and the secondary aims, with subthemes describing the findings in relation to respective theme (table 2).

**Table 2:** Summary of themes and subthemes derived from the data analysis, organised by study aims

Study aim	Theme	Subthemes included
Primary aim: assess healthcare professionals’ perceptions of the usability of the OPTIMAL AI tool (as proposed in a mock-up version), with a particular focus on data interpretation, practical implementation and the tool’s impact on shared decision making (SDM) in the consultation	Impressions of the content and appearance of the tool	Positive impressions Negative impression Ideas for improvement
	Perspectives on its potential application	Provides new insights into medications Guiding choice based on effectiveness Relevance to particular patients and consultations A spectrum of shared decision making Context of decisions guides approach Information is tailored to individual circumstances
	Practical considerations around implementation	Time Telephone consultations Medicolegal considerations
Secondary aim: uncover wider and more generalisable implementation aspects of AICDSSs	Interpreting the outputs from an AI-informed clinical decision support tool	Questions of trust Complements existing approaches Parallels to clinical experience Concerns of training-data quality Going against recommendations
Comparing the responses between GPs and NPPs: post-hoc observations from the above results.		

AI, artificial intelligence; AICDSSs, artificial intelligence-based clinical decision support systems.

Below, the results are presented in relation to the two aims with the corresponding themes. Subthemes are being explored under these themes, alongside a selection of direct quotes which reflect the range of participant opinions.

## Primary aim: Healthcare professionals’ perceptions of the usability of the OPTIMAL AI tool

### Impressions of the content and appearance of the tool

Most participants shared positive impressions of the tool, describing that it contained an appropriate level of relevant data. Only one felt there was too much information to use in a primary care consultation. Overall, it was felt to be simple, clear and visually appealing. Specific references were made to the value of including a ‘number needed to treat’ and ‘number needed to harm’ by both GPs and NPPs, as well as including the estimated outcomes for doing nothing (ie, not altering medication), in particular with regard to all-cause mortality:

“When people look at things like three percent mortality, they would be like “Oh I want zero percent mortality”. But you’ll be like “No, what it means by this is that obviously there is always a risk of mortality with any type”. What I probably would have liked is maybe a square saying what happens if you did nothing.” GP #4

Perceived limitations included the tool being limited to pharmacological treatments (important in the context of depression as used in the vignette: eg, no data on psychological therapies), and that if presented at face value, some of the data may appear alarming to patients who, for example, may not expect an increased risk of mortality:

“At the moment I wouldn’t show them the last tile which is the mortality stuff because I think that would scare the living daylights out of them especially if you said there’s a six percent risk that you’re going to die plus a three percent risk of stroke.” NPP #3

All participants contributed to ideas for further developing the tool, including requests to include drug doses and to include clinical guideline along the tool’s recommendations. While not the primary role of the tool, several participants considered the brief summary of patient characteristics (eg, co-morbidity, vital parameters and demographics) to be the most useful part, representing an unmet need in the existing electronic health record (EHR):

“Getting an overview of a patient from the EHR and actively looking for things is very time consuming. So, something like this tool which extracts that data for you, that is able to identify what is going to be crucial to your decision-making process, having that there as a table just takes out all the clutter [of the current EHR].” NPP #5

### Perspectives on applications and shared decision making

Most participants described new insights into the potential effects on long-term co-morbidities of the medications described in the output data. Insights included the predicted extent of change in existing conditions, such as blood sugar control in diabetes. Some described this as leading them to rethink their initial impression of the optimal medication to recommend. They also appreciated the measures of effectiveness of the different medications (percentage of patients who improved in a year and the expected quantitative change in other parameters, eg, a 2 mmol rise in HbA1c).

“I wasn’t sure whether sertraline would do this or do that. If there is a drug that I think well that’s really going to push her HbA1c up and we’re already on the cusp of being too high, that’s amazing that this technology can give me that insight.” NPP #2

Further perceived benefits included that the OPTIMAL tool could prevent ‘locking in’ on a certain medication due to habit, which could encourage lateral thinking from the prescriber:

“I think the joy of [a tool such as OPTIMAL] is that it might bring to the fore ideas that are not necessarily at the forefront of your brain.” GP #1

However, concerns were raised whether the tool was suitable for shared decision making, or whether it instead may complicate a patient-focused consultation:

“I think a lot of patients often don’t want this level of information and they don’t want to be that involved in a decision like this. I think they just want to be told ‘these are your options, this is how they’re going to help you, these are the big risks’ and that’s it and they’re happy to then just say ‘OK, well if that’s what you think I’m happy to go ahead with it” GP #2

In the above quote, the interviewee was alluding to wider questions about the role of the clinician when using CDSS, whether they are derived from large datasets and AI (such as the OPTIMAL tool) or from clinical guidelines, and how they impact SDM and the professional role of the prescriber. The degree to which OPTIMAL could be used in SDM was described by some participants as being influenced by the complexity of the decision or by the level to which the patient wished to guide it. In practice, all participants expressed that the OPTIMAL tool might be more suited to use in some consultations over others. This included matching patient preference for information. The outputs might also be used where clinical guidelines do not present a single recommended treatment.

“[Through] giving a figure to the patient, they could understand what we’re getting to, so the expectations are matched. And then reassuring them as well that a new prescription doesn’t interact with their medication…” NPP #1

The above was also reflected in its particular context of MLTC. Participants recognised the context of the case vignette presented in their own practice, where they needed to consider the relevance of other chronic conditions. The limitations of current clinical guidelines were highlighted, as well as the growing number of available medications.

“And I think that’s where this interests me, that we’re suddenly faced with a plethora of [diabetes] drugs and injectables. So, I see that maybe AI could help me there, guide me into what’s the best choice for the patient when they’ve got other chronic diseases” NPP #2

Participants also emphasised that the tool may serve certain, more engaged, patient populations better than others:

“I think patients who have taken more of an interest in their health and have done reading about various diseases or have specific worries as well. I think probably more highly educated patients as well take more of an interest.” GP #5

“It depends on how involved the patient wants to be in the decision-making process. For instance, if you’ve got a younger patient, so someone in their twenties or thirties or forties, I think they engage quite well.” GP #7

The above quote mirrors our findings in relation to SDM, that certain patient groups are more enthusiastic about SDM than others. This could be as some patient groups may be more able, motivated or empowered to engage in SDM than others and already undertake active involvement in their own care, with or without a CDSS. Finally, several GPs expressed the opinion that SDM in practice can be a codeword for convincing the patient to follow the narrative of the health professional, rather than actually involving the patient in their care:

“[CDSS] are quite useful if you want them to understand or maybe want them to be compliant towards it, or maybe if you’re just about starting them on something.” GP #8

### Practical considerations around implementation

The importance of integration of a new tool into existing clinical systems emerged as a key theme across almost all interviews. Existing integrated tools were viewed positively while having to input patient data into any new tool manually would significantly limit its appeal and potential uptake.

“I think you have to be honest really and it would depend how long it would take and how clunky it would be to use, so the information is great, but the actual practicalities of using it, that would mean whether it would be used or not I think.” NPP #4

Such integration was also seen as essential in order to prevent confusion with regard to patient selection. If running as separate computer application, there may be a risk of the AICDSS displaying data derived from another patient than the one managed in the EHR, leading to a risk of incorrect clinical management and ultimately harm.

Integration was also raised by some in relation to accountability, questioning whether or not the data generated from the tool were stored with the patient record. Being clear about what information informed a decision, regardless of how exactly it was weighted, was seen to be important.

“But quite a big deal about it that we forget is the accountability and we’re accountable for our actions and at the end of the day, it comes to justifying – rather than ‘why did you get it wrong?’ it’s ‘what did you do?’ because you could get things wrong but as long as you justify, you had good reasons, with a good will, and there’s no lack of duty of care I think that’s fine. That’s why I think it’s important to be linked to the patient medication record in some sort.” NPP #1

However, the introduction of more tools and guidelines could also be problematic when faced with choosing whether to use an AICDSS or not:

We are ultimately responsible and then it’s like “are we then more liable for not using the tool that’s been given to us, especially if the tool is actually giving you good evidence?” GP #4

Most participants emphasised the need for a practically useful AICDSS to work within the time constraints inherent to clinical practice. There was a widespread concern that an AICDSS, while potentially useful, would not fit into the existing workflow as it would require an extra step in the consultation. Interviewee #8, a GP, mentioned that such implications may necessitate a choice of either AICDSS or clinical guidelines to be applied, not both. Such action would not be coherent with the intention of the OPTIMAL tool, to complement rather than replace clinical guidelines (this potential conflict is also discussed later):

“I don’t know if it would be feasible to try and do all of those things in one appointment for that one patient. I think you’d almost have to go in choosing one, either you’re going to use OPTIMAL or you’re going to go guidelines based on this appointment, and then decide like that. I think personally I would find it quite difficult to have the NICE guidelines and have this up and have a discussion with the patient and come to a conclusion in ten minutes.”

In addition, the growing use of remote consultations was highlighted. Many medication reviews were reported to be conducted via telephone, precluding opportunities to directly share the outputs from the tool with patients:

“With telephone consultations, that a lot more now, as our workload’s increasing, you’re doing telephone consultations, so you don’t have that opportunity either.” NPP #2

To summarise, participants were largely positive to the prospect of using the OPTIMAL tool in their practice. Potential advantages included prompting the clinician to ‘think outside the box’, help with optimising prescription in patients with MLTC, and for a subset of particularly receptive patients, to aid SDM. Perceived disadvantages included the tool taking valuable clinical time, potential issues with accountability in case of clinical harm, and how the tool could be integrated into remote consultations.

### Secondary aim: Considerations on interpreting the outputs from an AICDSS

To explore the secondary aim, we collated responses relating to the interpretation of outputs from AICDDs in general. During the introduction to the OPTIMAL tool, the nature of the underlying data source and the way the data were generated was explained to the participants. Some participants questioned whether they would trust the outputs and sought reassurance that the tool would be trialled before its implementation.

“I think it would be obviously very much confidence driven and that using something like this initially you would be cautious…” NPP #2

Participants also raised concerns about the fundamental approach of basing an AICDSS on historical clinical data (such as OPTIMAL being based on CPRD Aurum data), as data quality may be compromised due to the manner in which it was collected as well as the actual actions of the clinician inputting the data in the first place:

“I think what concerns me slightly is how trustworthy the original data is. Because if it’s based on decisions that doctors have made that maybe weren’t necessarily the best decisions or maybe not always great documentation. I know how most people document consultations about depression, it’ll be a bit of sort of “four weeks after starting sertraline patient feels they’re picking up a bit” – how is that transferred into then an actual comparable data point?” GP #2

Ultimately, almost all participants reflected on the primacy of their individual judgement, particularly if presented with data that contradicted their own clinical experience. In such circumstances, some reported they would go back to more basic principles to sense check the data being presented. Some reflected that this also came with increasing experience, and a potential risk was less experienced clinicians following the recommendations of the tool too closely.

“Yeah, so it does all the work and then makes recommendations and then that’s where the human intelligence comes in I would say, so the human decides whether to accept the recommendation or adjust it.” NPP #9

GP #10 argued that it would be difficult to go against the recommendations provided by the tool, even if the prescriber intuitively wanted to do so:

“I would struggle to go against it, especially if fluoxetine is not my first line, you know, for depression we don’t go for fluoxetine, so it would be a bit negligent to do something like that I think, if it’s showing you a risk and you’re going for duloxetine when mirtazapine and fluoxetine are better options then, I’d do as the tool says. Because now you’re going to get into a territory where you’ve also got more information for patients to maybe even say go down the medicolegal route.” GP #10

In this quote, the participant expresses an assumed risk of going against the tool, fearing that it may be viewed as superior to a human prescriber in a medicolegal situation much in the same way as clinicians would likely be hesitant to prescribe against the pharmaceutical company’s declared contraindications. Some participants also drew a parallel between the insights from real world primary care data and their own clinical experience, reflecting on the accumulation of clinical experience of similar patients.

“I think as a clinician, I can only speak for myself, I think even our decision-making process is observational. We accumulate that data from previous experiences with patients.” NPP #5

Another participant compared the use of an AICDSS to having a clinical meeting with colleagues, discussing cases but ultimately making your own judgement:

“It’s almost like, you know, we talk about artificial intelligence and human intelligence, but it’s almost like two colleagues sitting together, like a clinic meeting, you’re never going to all come up with the same outcome, but you’ve still got your own clinical judgement.” GP #9

Most felt that the tool could complement existing sources of information for prescribing decisions and some reflected that the tool would act like a second opinion, either informing or validating their own thought process.

“To me it’s just like discussing with a colleague a case – one person has one view and you have another and it gives you room to think about what you’re doing.” NPP #9

However, combining different sources of information such as clinical guidelines and the OPTIMAL tool outputs was seen as challenging by several interviewees, in particular when they provided conflicting advice:

“I think at the moment if the guideline is different than this, it would be quite complex in that single appointment, to try and look at the guidelines, decide on that, look at this, decide on that, and see what the patient wants. I don’t know if it would be feasible to try and do all of those things in one appointment for that one patient. I think you’d almost have to go in choosing one, either you’re going to use OPTIMAL or you’re going to go guidelines based on this appointment, and then decide like that.” GP #8

GP #5 expressed this potential conflict as a clinical risk, where the OPTIMAL tool may promote a potentially contraindicated medication to the clinical guidelines, due to not having the inbuilt capability to check against them. In this situation, it may be difficult for the clinician to weigh the OPTIMAL tool against clinical guidelines:

“You could be in a situation where one drug looks really good but is contraindicated for that patient. And you might have not thought of that drug because you just “Oh I don’t use it in those kind of patients” but then this goes “Oh have you thought of – why don’t you use trazodone” or something. You could inadvertently push people into prescribing things that they don’t usually prescribe without them really thinking why they don’t usually prescribe it, and there might be good reason.” GP #5

On the contrary, one participant expressed the view that this likely would not be an issue if the tool included an explanation of the data sources used:

“I don’t see it as like a negative point. It’s just to bear in mind that OPTIMAL is coming from observational studies rather than clinical trials, so if there is something there that doesn’t fit in with the general picture, I might have to give it careful consideration.” NPP #1

This interviewee, as such, expressed confidence that clinicians would be able to simultaneously interpret and make decisions based on personal knowledge and experience, clinical guidelines and recommendations from the OPTIMAL tool.

In summary, participant views differed on whether the way that the OPTIMAL tool and similar AICDSSs are constructed (ie, based on large datasets of historical clinical data) was cause for concern, and how to interpret outputs from the tool in comparison to making reference to clinical guidelines and clinical trials. Potential issues included lack of trust in the performance of the tool due to perceived poor training-data quality and how to interpret the outputs of the tool when they were in disagreement with either clinical guidelines or personal knowledge and experience.

### Post-hoc comparison between GPs and NPPs

As stated, this study was not designed to compare the views of different professions. However, some broad observations can be made. NPPs were in general more positive about the OPTIMAL tool than GPs, expressing few concerns about its use case and impact. This was also seen in some of the descriptions of seeing the tool in a positive role as a teacher. In contrast, several (but not all) GPs were sceptical about using the OPTIMAL tool in their clinical practice, citing concerns about implementation, safety and accountability. NPPs as a group were also more enthusiastic about using the tool for SDM. This may reflect a different patient-clinician relationship and different approaches to decision making in prescribing practice between the two groups.^21^ Furthermore, this may also be reflected in potentially different information needs, with pharmacist participants suggesting scope for inclusion of further information on doses and durations, while the lack of information on psychological therapies may be more less relevant to this group.

## Discussion

### Findings in context

This study explored prescribing clinicians’ perceptions of an AICDSS to support prescribing decisions in the context of MLTC. The majority of interviewees saw benefits of the presented OPTIMAL tool. These included expected benefits such as more individualised prescribing, reduced risk of prescribing error, and the tool providing a platform for SDM (although views differed on what SDM entailed). Further beneficial aspects included the ability for the tool to trigger the clinician to think outside the box and to reflect on the effects of pharmaceuticals on multiple co-morbidities, in contrast to habitually prescribing a certain medication (eg, sertraline for depression). Interestingly, interviewees who were more generally positive about the tool commonly described its use case as that of ‘a teacher’ or ‘a colleague’ acting as a guide to the clinician and providing new perspectives in the consultation. Other interviewees viewed the OPTIMAL tool and AI in general in a more authoritarian manner: a know-it-all imposed on them to ultimately replace them, without considering the more holistic aspects of the patient consultation. Holford (2020),^22^ for example, elaborates on this from a sociological perspective, discussing how increased digitalisation and ultimately digital technology as actors (ie, taking an active part in shaping the care) in the patient consultation risks downplaying the ‘soft’ values of providing healthcare: the preferences of the patient, the ‘gut feeling’ of the clinician and the therapeutic effect of the consultation itself.

Several interviewees also found the relationship between the OPTIMAL tool and established clinical guidelines problematic. In the hypothetical situation that the recommendations from these two sources would not align, interviewees were conflicted on how to value and compare the different sources. AICDSSs have the potential to bring unique benefits compared with clinical guidelines: through relying on very large datasets, they are able to provide individualised recommendations, taking into account the complexities of MLTC and differing demographics in a manner that a clinical trial or single guideline cannot do. However, these systems derive their recommendations through what is essentially a retrospective cohort study, using large clinical data registers of past patient cases to predict the outcome of a certain treatment. Because of this, they are fundamentally unable to base their predictions on principles of causality: for example, patients may gain weight because of certain medications, but it could also be that certain medications are typically given to a patient population who are already prone to gaining weight. Guidelines on the other hand tend to be based on clinical trials, where there is a higher burden of proof of causality, as well as incorporating practical, ethical and health-economical considerations. Communicating this to users of AICDSS through provision of accurate, accessible and tailored information and training would support development of confidence and proficiency to accurately judge different information sources against each other. This could be achieved by e-learning training and through integration of AICDSS training in the curriculum for HCPs in training. Where issues arise in applying potentially conflicting information, transparency in what data a recommendation is based on is important, and to the extent possible, how the AI made the recommendation. In a wider perspective, this also highlights the importance of transparency and regulation in AI development and implementation at large. Research in this area is urgently needed.

### Preceding scholarship

Blease et al (2019)^23^ conducted a study on GPs and compared their views to those of bioinformaticians. They found widespread scepticism around the usability of AI, and concerns around accountability and biased algorithms among others, but commonly expressed a belief that AI could be of benefit to general practice and may form part of the solution to workforce problems. Ganapathi and Duggal (2023)^24^ found similar themes in their interview study of UK GPs and emphasised the need for training of prescribers in order for them to safely apply AI in practice, while Buck et al (2022)^25^ interviewed German GPs and found a hope that AI would bring increased efficiency and accuracy, but feared for the loss of human involvement and medicolegal implications. Concerns of lack of human interaction and subsequent personalisation of interventions were reflected in a recent interview study with MLTC patients and their carers,^18^ but likewise, AI was seen as potentially very useful in navigating complex clinical and social needs. Finally, Oremule et al (2024)^26^ interviewed UK GPs and found similar results while raising risks of algorithmic bias: Numerous precedents exist where AI-based or big-data-based CDSSs have been found to produce different accuracy and effectiveness for different demographic groups, potentially widening health inequities.^27^

### Strengths and Limitations

In comparison to abovementioned existing studies, ours was conducted alongside the development of a tool with a long-term aim to be developed for clinical practice, sets it apart from preceding scholarship which has mainly explored views on hypothetical AI applications. Further, we add to the previously explored themes the potential conflict between clinical guidelines and clinical intuition vs AICDSS guidance as an area in need of exploration.

The study gathered the perspectives of a range of participants of different clinical backgrounds and demographics but was not set up to robustly compare differences by these characteristics and is limited to brief observations of interprofessional differences. However, referring to the abovementioned concept of information power, we are confident that we have captured perspectives largely reflective of West Midlands primary-care clinicians as a broad group. While our results are likely to be applicable beyond this geographical area, differences in local EHR systems, prescription policies, patient population and clinical pathways mean caution is required when extrapolating. However, the proposed OPTIMAL tool as studied is not system-specific. In addition, the majority of the interviewees had previously participated in a related, currently unpublished, interview study on the views of AI in MLTC in general. This may have affected their answers in our study. For example, the reflections and discussions in the preceding study may have made them more positive towards AI in general, or they may have withheld answers from our interviews as they felt that they had already covered some topics in the preceding interviews.

### Implications for research and practice

Widespread implementation of AICDSSs in primary care is well underway, and the pace of innovation is predicted to increase.^28^ However, the implementation of medical technology such as CDSSs is known to be a complicated process taking place in the highly complex setting of pre-existing clinical practice.^7^ With this in mind, we undertook this study to better understand the context in which the OPTIMAL tool is to be implemented, and the findings will help to optimise the tool and its implementation process. Beyond OPTIMAL, the views, and the perceived benefits and issues uncovered in this study are largely applicable to a wider context of AICDSS in healthcare and will help guide the development and implementation of AICDSSs.

Further research is needed into how to achieve successful implementation of AICDSS in primary care, with a particular focus on the role of AICDSSs in the decision-making process of the clinician, in order to realise the full potential of them. Such research may, for example, apply established implementation-science-derived approaches such as Normalisation Process Theory^29^ or derivatives thereof^30^ to understand how a given intervention can be made compatible with the existing clinical context, including how AICDSSs can be used and interpreted alongside existing clinical guidelines. Additionally, further exploration of differences in the perspectives of clinicians from different clinical backgrounds would help to better understand how information needs and approaches to successful implementation may need to be tailored to different groups.

## Conclusion

The OPTIMAL tool (and similar AICDSSs) holds potential for optimising pharmaceutical treatment in patients with MLTC, through more individualised consideration of the impact on current and future conditions. However, specific issues including data interpretation and overlap with other prescribing guidance need to be addressed with careful implementation into the existing clinical practice necessary to realise the potential benefits.

## Supplementary material

10.1136/bmjopen-2025-102833 online supplemental file 1

10.1136/bmjopen-2025-102833 online supplemental file 2

## Data Availability

Data are available upon reasonable request.
