# Supplementary material for: Perceptions of an AI-based clinical decision support tool for prescribing in multiple long-term conditions: a qualitative study of general practice clinicians in England
Source: BMJ Open. 2025 Nov 23;15(11):e102833. doi: 10.1136/bmjopen-2025-102833 (PMC12645610; doi:10.1136/bmjopen-2025-102833)
Supplement: online supplemental file 1 [file bmjopen-15-11-s001.docx]

 
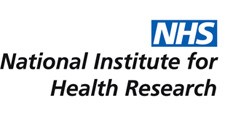


 
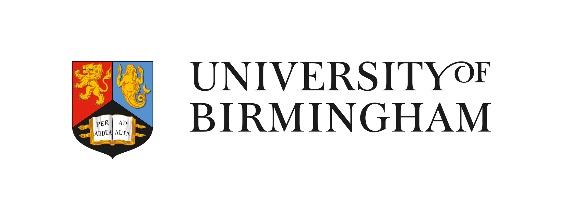


**OPTIMIsing therapies, disease trajectories, and AI assisted clinical management for patients Living with complex multimorbidity (OPTIMAL study):**

**Participant information sheet: staff interviews**

You are being invited to take part in an interview as part of a research study.  Before you decide if you are willing to be interviewed it is important for you to understand why the research is being done and what it will involve. Please take time to read the following information carefully and discuss it with colleagues if you wish. Ask us if there is anything that is not clear or if you would like more information.

**What is the purpose of the study?**

The study aims to use artificial intelligence (AI) to produce computer programmes and tools that will help improve the treatment and choice of drugs in patients with clusters of multiple long-term conditions (complex multimorbidity or cMM).

Artificial intelligence (AI) is a computer system that can conduct tasks that would normally need human intelligence. There are examples of AI being used in our everyday lives, with applications and software such as Spotify, Amazon and BBC iplayer. These ‘apps’ and on-line websites function by predicting what kind of music, TV programmes, or general purchases we may like or want. They do this by using a computer programme that can observe what categories and kinds of TV, Music etc. we watched or bought in the past and use this data to predict what we are a likely to choose in the future.

The machine learning branch of AI is also used in things like self-driving cars, language translations and it is being developed for use in healthcare. Newer AI methods makes it easier to process large amount of health data in a short time. These AI methods can give doctors and patients information that will help improve the care of people with four or more long-term health conditions.

AI can be used in healthcare to help guide the diagnosis of long-term health conditions, plan the best treatment strategies, and predict the next health condition that people might develop. This is especially relevant for people who have several different health conditions because the guidelines that doctors and healthcare professionals use for one condition does not usually consider other health conditions.

AI-based decision-making tool for managing multiple health conditions may help patients and healthcare professionals make more well-informed shared decisions. Patients and healthcare professionals may see many benefits to the use of AI in healthcare. However, they may be concerned this could be harmful or that it could affect the relationships between healthcare professionals and patients. We want to speak to patients and healthcare professionals to understand their views about AI in healthcare. It is important in healthcare research that we reflect these experiences in any future plans for the use of AI in a healthcare setting.

Data collected from this study will give new insights to how healthcare professionals and people with  four or more long-term health conditions view AI-based decision-making tools. It will also tell us which factors in the computer program are important to them and what options they prefer. This information will also be used to make AI better to use in practice.

**How do we plan to do this:**

1. By linking large, detailed databases of Electronic Health Records (EHR) of patients who attend GP services and hospitals that have been collected since 1999. These records include patients' diagnoses, medications, blood tests, readings such as blood pressure, scans, and specialist tests. By using artificial intelligence (AI) methods, we hope to be able to show how the different mixes of diseases arise over time. It should also help us better understand how different drugs may interact with other drugs.
2. By asking people with four or more long-term health conditions and health care professionals about their knowledge and views about using AI to make decisions about health care.
3. By using AI techniques to combine data, and together with the input from people with multiple long-term health conditions and health care professionals, to develop a Patient Similarity Tool. This will predict which drug we should give and when we should give it to someone to reduce the risk of harm and bring about maximum benefit. It will also tell us what disease people may get next.
4. By examining the best way to present information in the computer program to people with multiple long-term health conditions and health care professionals by asking them about what is important to them and what options they prefer.

Our team includes patients, public members, and world leading experts from UK universities with expertise in biology, AI, medicine, health service research, public health, and general practice. Working as a multidisciplinary team we hope to improve the health and care for people with multiple long-term health conditions

**Why have I been asked?**

You have been chosen because you are a healthcare professional working in primary or secondary care within the West Midlands region with experience caring for people with cMM. We aim to interview 30 healthcare professionals on total.

**Do I have to take part?**

Taking part in the study is completely optional, the decision to take part is entirely up to you. Whether you decide to take part or not will have no effect on your job and you can leave the study at any time, without giving a reason. If you decide to take part we will ask you to sign the consent form at the end of this sheet. If you decide to take part you are still free to withdraw at any time and without giving a reason.

**What do I have to do?**

We are asking you to be interviewed by a qualitative researcher at a time and place that is convenient to you. The interview will be conducted either face-to-face or via telephone or video call. We expect each interview to last up to an hour. The interviews will be audio recorded on to a secure audio recording device. The recording and transcript will be kept completely confidential using a study ID code and only the University evaluation team will have access to them

At the end of the interview, you will be asked if you would be willing to be contacted to take part in a follow-up interview. This will be to determine factors that are important in relation to the how the results of the patient similarity tool that is being developed are presented.

**What are the possible risks and disadvantages of taking part?**

Taking part in the interview will take some of your time. However, you can choose a time and date that is suitable for you. In light of COVID-19, we will adhere to current government guidelines and local procedures in order to minimise the risk of exposure. . Support for participants will be provided to set up an online conferencing link, or by telephone according to participant preference. Researchers will have evidence of vaccination and/or negative results from a lateral flow test prior to interview if face-to-face.

**Will I receive any financial reimbursement for taking part?**

You will be offered a £15 Amazon voucher as a thank you for taking part in the interview.

**Will my taking part in the study be kept confidential?**

All information collected about you for this study will be subject to the General Data Protection Regulation and Data Protection Act 2018 for health and social care research and will be kept strictly confidential.

All audio-recordings will be kept for 10 years after the end of the study and then destroyed. Documentation and data from this part of the study will be securely stored at the University of Birmingham for 10 years.

**How will we use information about you?**

The University of Birmingham is the Sponsor for this study and this means that the University of Birmingham are responsible for looking after your information and using it properly. University of Birmingham will keep identifiable information about you for at least 10 years after the study has finished, to allow the results of the study to be verified if needed. Information collected from you will be used for this research project. This information will include your:

- Full name
- Telephone number
- Gender
- Age range
- Job role
- Years since qualification

People who do not need to know who you are will not be able to see your name or contact details. Your data will have a unique study number which will be stored separately from any potentially identifiable data (job role, gender, years since qualification) and all information will be kept safe and secure. In the research team, you will be identified using your unique study number.

All information collected by the Sponsor, including a copy of your signed consent form, will be securely stored at the research study office at the University of Birmingham on paper and electronically and will only be accessible by authorised personnel. The only people in the University of Birmingham who will have access to information that identifies you will be people who manage the study or audit the data collection process.

The audio recordings from the interview will be transcribed by a transcription company which has been approved for transcription of medical data. If you agree to take part in the interview study, your name will not be on the recording and we will remove your name from the interview transcripts to keep your identity confidential. Direct quotes may be used in publications but these will be numbered and anything that could identify you will be removed. Nothing that you say will be fed back to the doctors and nurses involved in your care as coming from you.

**What are your choices about how your information is used?**

You can choose to stop taking part in the study at any time, without giving a reason, but we will keep information about you that we already have. If you agree to take part in this study, you will have the option to take part in future research using your data saved from this study. To safeguard your rights, we will use the minimum personally identifiable information possible. You can find out more about how your information will be used at <https://www.birmingham.ac.uk/privacy/index.aspx>.

The University of Birmingham will use your name and contact details to contact you about the research study, and make sure that relevant information about the study is recorded and to oversee the quality of the study. Individuals from the University of Birmingham and regulatory organisations may look at your research records to check the accuracy of the research study.

All individuals who have access to your information have a duty of confidentiality to you. Under the provisions of the General Data Protection Regulation (GDPR) 2018, you have the right to know what information the Trial Office has recorded about you. If you wish to view this information, or find more about how we use this information, please contact the University of Birmingham’s Data Protection Officer at the address below.

**Where can you find out more about how your information is used?**

If you would like more information on your rights, would like to exercise any right or have any queries relating to our processing of your personal data, or if you wish to make a complaint about how your data is being or has been processed, please contact:

**The Data Protection Officer, Legal Services, The University of Birmingham, Edgbaston, Birmingham B15 2TT
Email: dataprotection@contacts.bham.ac.uk
Telephone: +44 (0)121 414 3916**

You can also find out more from [www.hra.nhs.uk/information-about-patients/](https://www.hra.nhs.uk/information-about-patients/) and by reading the information available here [**www.hra.nhs.uk/patientdataandresearch**](http://www.hra.nhs.uk/patientdataandresearch)**.**

**What will happen if I don’t want to carry on with the study?**

Your participation is completely voluntary. If you choose to take part but change your mind later, you are free to leave the study at any time, without giving a reason, and without your employment or legal rights being affected. If you wish to withdraw from the study, please contact a member of the team (details are at the end of this document), However, please note that if you decide to withdraw more than 2 weeks after participating in an interview, any data already collected may still be used in the study.

**What happens next?**

If you would like to participate in an interview, please contact a member of the research team whose details are given below. You will have the opportunity to ask further questions, to decide if would still like to participate. *Please note that because we would like to interview a range of people depending upon certain characteristics we will ask you about your job role, length of practice, age gender, ethnicity when you contact us. Following this it may take a number of weeks before we contact you to arrange an interview. In the event that we do not require your assistance, we will contact you to let you know.*

**What will happen to the results of the research study?**

The information we collect will be analysed and the results will be presented in several ways:

- A short written summary of the results will be available on the OPTIMAL website, or will be emailed to you upon request
- A detailed report will be written and will be available upon request
- We will publish the results in academic journals.

Your details will not be shared at any time and you will not be identified in any of the results from the research.

**Who is organising and funding the research?**

The evaluation is funded by the National Institute for Health Research (NIHR). The research is sponsored and insured by the University of Birmingham.

**Who has reviewed the study?**

All research in the NHS is looked at by an independent group of people called a Research Ethics Committee (REC), to protect your interests. This study has been reviewed and given favourable opinion by South Central – Hampshire B Research Ethics Committee (REC Reference: 22/SC/0210). Patients and public Involvement (PPI) members have been involved throughout all stages of the research study.

**What happens next?**

If you would like to participate in an interview, please contact a member of the research team whose details are given below.

**The research team:**

**OPTIMAL Team**

**Institute of Allied Health Research,**

**University of Birmingham**

**B15 2TT**

[**J.cooper.5@bham.ac.uk**](mailto:j.cooper.5@bham.ac.uk)


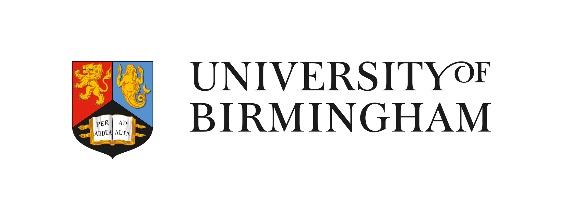


**
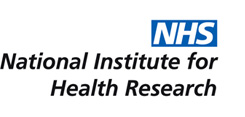
**

**OPTIMIsing therapies, disease trajectories, and AI assisted clinical management for patients Living with complex multimorbidity (OPTIMAL study)**

**Participant study ID: _________**

**Consent form: Staff Interview**

**Please initial box**

1. I confirm that I have read and understood participant information sheet version 1.1 (dated 18.07.2022) for the above study. I have been able to consider the information, ask questions and have had these answered satisfactorily.
2. I understand that my participation is voluntary and that I can withdraw up to 2 weeks after the interview without giving any reason and without my employment or legal rights being affected. I understand that if I decide to withdraw from the study after this time, any data already collected may still be used in the analysis.
3. I understand that relevant sections of my data collected during the study, may be looked at by individuals from the University of Birmingham, from regulatory authorities or from the NHS Trust, where it is relevant to my taking part in this research. I give permission for
   these individuals to have access to my data.
4. I agree to take part in an interview with a member of the research team

for this study.

1. I understand that the interview will be audio recorded, and that the recording will be securely stored. I agree to my interview being audio recorded by the research team
2. I understand that a written record of the interview will be made by a transcription company contracted by the research team, but neither my name nor anything else that identifies me will be used in the written version.

I understand that the researchers might use my words in their reports or publications, but this will not be done in a way that identifies me.

1. I would like to receive a copy of the study report YES/NO
2. I agree to be contacted by a member of the team regarding the second stage of the study.
3. I have received a physical copy of the consent form and Participant Information sheet for my records (or provided my email address to receive an electronic copy.
4. I agree to take part in this study.

| **Name of participant Signature Date** |
| --- |
|  |
| **Name of researcher Signature Date** |
|  |

One copy for the participant, one for the participants medical record, one original for the study folder.
