# Supplementary material for: Perceptions of an AI-based clinical decision support tool for prescribing in multiple long-term conditions: a qualitative study of general practice clinicians in England
Source: BMJ Open. 2025 Nov 23;15(11):e102833. doi: 10.1136/bmjopen-2025-102833 (PMC12645610; doi:10.1136/bmjopen-2025-102833)
Supplement: online supplemental file 2 [file bmjopen-15-11-s002.docx]

Shared decision making:

- Do you use any information as part of the shared decision-making process?
  - How?/ What information?
- Do you use a computer as part of the shared decision-making process?
- How often do you use it? Most of the time or sometimes? When?
  - How do you use your computer and screen?
  - Do you share information on your computer screen with the patient? How?
  - What are the barriers to this? (e.g. if patient can’t see the information on the screen).
- Do you have any experience of using risk predictive tools in shared decision-making/ as part of a patient consultation? E.g. QRISK, Centor, FeverPain.

Preferences for types of information on risk presented for shared decision-making.

**Imagine that you have a consultation with this patient** *(present case vignette)* **and will be making shared decisions about management of the conditions and treatments.**

These are the kinds of information about risk that the AI tool will create for use within the consultation [*Show mock-up of information generated by automated risk assessment*].

- How would you feel about using this information to inform shared decision-making as part of a consultation with patients with four or more long term conditions?
- How would you use the information in practice?
- What would influence your decision to use the information?
- The information will mainly be from observational studies rather than RCTs – would this affect whether/ how you would use this information? Why?
- Are there elements that you would find more or less relevant?
- What would make you more/ less likely to use the information? (probe for time issues?)
- Do you think you would need to explain how the data was generated with the patients?
- How would it change your interaction with the patient?
- Are there examples where you would be more/ less likely to use the tool?
  - Would it need to be prepared forhand? Would your practice have resources for that?
- Would you show the information to the patient by turning your computer screen?
  - How?
  - Why?
  - What challenges might there be?
- Do you think that using the information would change the consultation? Why?
- Are there some groups of patients that you would be more/ less likely to use the tool with? Probe for continuity of care issues (e.g. depending on how well you know the patient)

Preferences for look and feel for how the information is presented

We will present to the participant with an example of the kinds of risks the tool will generate and how the information might look.

**Imagine the computer algorithm produces this infographic for this patient.**

- Do you think that you would use the infographic as part of the shared decision-making process in the consultation with this patient? Why?

***If not*** – ask

- What might be more useful for you?

***If yes*** – probe for

- How would you use the infographic?
- Which part of the infographic is most helpful? Why
- Which parts are less helpful?
- What other information would be helpful?
- Do you have any preferences for how the information should be presented? Look and feel
- Would you show the infographic to the patient?
  - Why?
  - What challenges might there be?
- Do you think that using the infographic would change the consultation? Why?
- Are there some groups of patients that you would be more/ less likely to use the tool with?
  - Why?
- Would the practice of others affect your use of the tool? Would there be particular groups of professionals who might use the tool more / less than others?
- What would you do if you disagreed with the output from the tool?
