# Supplementary material for: Perceptions of an AI-based clinical decision support tool for prescribing in multiple long-term conditions: a qualitative study of general practice clinicians in England
Source: BMJ Open. 2025 Nov 23;15(11):e102833. doi: 10.1136/bmjopen-2025-102833 (PMC12645610; doi:10.1136/bmjopen-2025-102833)
Supplement: Supplementary file 3 [file bmjopen-2025-102833.reviewer_comments.pdf]

PEER REVIEW HISTORY

BMJ Open publishes all reviews undertaken for accepted manuscripts. Reviewers are asked to complete a checklist review form and are provided with free text boxes to elaborate on their assessment. These free text comments are reproduced below.

ARTICLE DETAILS

Title (Provisional)

Perceptions of an AI based clinical decision support tool for prescribing in multiple long-term conditions: a qualitative study of general practice clinicians in England

Authors

d'Elia, Alexander; Morris, Simon George; Fitzsimmons, Leah; Hathaway, Ellie; Cooper, Jennifer; Nirantharakumar, Krishnarajah; Jackson, Thomas; Marshall, Tom; Jackson, Louise J; Crowe, Francesca; Haroon, Shamil; Greenfield, Sheila

---

VERSION 1 - REVIEW

**Reviewer**	**1**
**Name**	**Ouanes, Khaled**
**Affiliation**	**Saudi Electronic University, College of Health Sciences, Department of Health Informatics,**
**Date**	**12-May-2025**
**COI**	**None**

Reviewer Comments on Manuscript ID bmjopen-2025-102833:

"Perceptions of an AI based clinical decision support tool for prescribing in multiple long-term conditions: a qualitative study of general practice clinicians"

Recommendation: Minor Revision

General Comments:

Thank you for submitting this timely and relevant manuscript to BMJ Open.

The paper is overall well-aligned with the journal's scope, and it obviously addresses an important and relevant research question using appropriate methods. And it presents valuable findings. There are no fundamental flaws.

This qualitative study provides valuable insights into the perspectives of general practice clinicians regarding the potential use and implementation of an Artificial Intelligence-based Clinical Decision Support System (AI-CDSS) for prescribing in the complex context of Multiple Long-Term Conditions (MLTC). Understanding these perspectives is crucial as such tools are

developed and integrated into practice, aligning well with BMJ Open's scope which includes qualitative research, health services research, and topics relevant to clinicians and patient care.

The study is well-designed, employing an appropriate qualitative methodology with semi-structured interviews clearly centered around a specific mock-up tool (i.e. OPTIMAL) and case vignette, which facilitated rich data collection. The manuscript is clearly written, the language is very good, the objectives are well-defined, and the thematic analysis is presented logically and supported by illustrative participant quotes.

I have to commend the authors for the thoroughness in reporting, including adherence to ethical procedures, appropriate use of descriptive statistics for participant demographics, and provision of supplementary materials like the COREQ checklist, which aligns with BMJ Open's standards for transparency and methodological soundness.

Overall, as previously established, this is a strong manuscript addressing an important area. I recommend Minor Revision to address a few points that I truly believe will further enhance the paper's clarity, quality, and impact.

Specific Points for Minor Revision:

Nuancing Interprofessional Differences: The observation regarding potential differences in perspectives between GPs and Nurse/Pharmacy Prescribers (NPPs) is quite interesting. While I understand the limitations imposed by the sample size, I encourage the authors to slightly expand the discussion on these observed differences. Even tentative explorations, carefully worded/nuanced, could add valuable insights regarding how professional roles might be capable of shaping perceptions and potential adoption of AI-CDSS tools.

Navigating Conflicting Guidance: A key strength of the paper in my opinion is highlighting the potential tension clinicians perceive between AI-CDSS recommendations (derived from observational data) and established clinical guidelines or their own clinical experience. To strengthen the implications, please consider elaborating further in the Discussion section on how clinicians might practically navigate such conflicts. Consider discussing the types of training, practice, support, or specific tool features (e.g., clear explanations of data sources and limitations, confidence scores for predictions) that might help clinicians critically appraise and appropriately weigh in different sources of information in their own decision-making process.

Practical Facilitation of Shared Decision-Making (SDM): The study provides a thoughtful exploration of the tool's potential role in SDM, noting varied views among clinicians. I recommend enhancing the practical applicability of the findings, by considering a slight expansion on how the OPTIMAL tool (or even similar AI-CDSSs) could be more effectively adapted, tweaked, customized, presented, or integrated into diverse clinical workflows (including remote consultations) to genuinely and practically facilitate SDM. This could

include discussing strategies to accommodate the varying levels of information desired by patients and clinician concerns about consultation time.

I appreciate the contribution this work makes to the field and look forward to seeing a revised version. This study clearly offers important considerations for the developers and implementers of AI-CDSS tools aiming to support primary care clinicians managing patients with MLTC.

**Reviewer**	**2**
**Name**	**Holt, Sian**
**Affiliation**	**University of Warwick Warwick Medical School, Division of Health Sciences**
**Date**	**27-Jun-2025**
**COI**	**None**

Overall, this is a very interesting and valuable paper which can help better inform future AI prescribing interventions. Consulting with key intervention users (e.g. GPs and other prescribers in primary care) is a useful methodology and likely to lead to an intervention that is more acceptable and feasible. It is also good to see that other papers are currently in preparation to consider the patient perspective.

From my review, I offer the follow feedback and suggestions for improvement:

1. Abstract – in your design, please specific the type of thematic analysis you used.

2. In your methods, it wasn't clear how the OPTIMAL mock-up used during your interviews was developed. You mention about stakeholder involvement but could be more specific on who was involved. I would expect to see a proposed theory of behaviour change or set of guiding principles, based on what is already known from the wider literature. This would then have informed the mock-up concept for your initial intervention. I recommend having a look into the Person-Based Approach to intervention development – you don't have to use this method, but hopefully this shows a way to clearly define how the intervention developed prior to this study.

3. I recommend reading the following paper (and other similar papers) that also use vignettes to discuss the use of AI clinical tools - Value of using artificial intelligence derived clusters by health and social care need in primary care: A qualitative interview study with patients living with multiple long-term conditions, carers and health care professionals

4. I think the methods section needs more work. You mention using an inductive thematic analysis but I wonder if this really fits what you have described. I recommend reading the Braun and Clarke article (Toward good practice in thematic analysis: Avoiding common

problems and be(com)ing a knowing researcher). There is a section around the difference between topic summary themes and meaning-based interpretive story themes which will be useful. I think your themes as currently presented as topic summary themes, and tie directly into your project aim/questions as your theme names/areas. This is fine (and very useful in pragmatic research) but potentially lends itself more to codebook thematic analysis than inductive thematic analysis e.g. you had a specific question/set idea of the things you were looking for within the data rather than being guided by narratives within the data. I think the methodology could be made clearer and be more detailed to enable replication of analysis approach.

5. "Themes were thereafter compared through consensus meetings and similar themes collapsed into each other." – In this sentence, I think you mean similar codes collapsed and not themes.

6. The themes within the text are not clear – your supplementary Table 3 presents your findings more clearly. I think you could find a way to describe your themes and subthemes using the format of your table.

7. Limitations – you reflect on the limitations of geographical area but could expand on this in terms of potential impact on implementation e.g. IT systems vary by practice and by region, complicating the ability to embed integrated, consistent tools

8. Limitations – you reflect on the quality of CPRD Aurum coding (which was reflected in your data). You could also reflect on the wider impact of AI tools on health inequalities within your limitations section

9. It was great to see your PAG mentioned and the input they had. I think you could provide some more specific information to strengthen this section e.g. how many members are in your PAG? Broadly, what lived experiences do they represent?

I hope you find these comments helpful. I think this topic and approach is worthwhile and wish you luck in the research.

VERSION 1 - AUTHOR RESPONSE

Reviewer comment	Author response	Amendments to text
Reviewer 1		
Nuancing Interprofessional Differences: The observation regarding potential differences in perspectives between GPs and Nurse/Pharmacy Prescribers (NPPs) is quite interesting. While I understand the limitations imposed by the sample size, I	Thank you for your interest in this finding. As referenced in the paper we intended to include a range of professionals in our sample but also recognise the limitations to draw robust	Results. Post-hoc comparison between GPs and NPPs<br>*"This was also seen in some of the descriptions of seeing the tool in a positive role as a teacher."*<br>…*"Furthermore, this may also be reflected in potentially*

encourage the authors to slightly expand the discussion on these observed differences. Even tentative explorations, carefully worded/nuanced, could add valuable insights regarding how professional roles might be capable of shaping perceptions and potential adoption of AI-CDSS tools.	conclusions given the resulting sample-size. We included a section post-hoc comparisons between professional groups. We have further reviewed and expanded in our results section to consider tentative explorations linked to our recommendation for future research.	*different information needs, with pharmacist participants suggesting scope for inclusion of further information on doses and durations, whilst the lack of information on psychological therapies may be more less relevant to this group."*<br><br>Discussion. Implications for research and practice. Para 2 *"Additionally, further exploration of differences in the perspectives of clinicians from different clinical backgrounds would help to better understand how information needs and approaches to successful implementation may need to be tailored to different groups."*
Navigating Conflicting Guidance: A key strength of the paper in my opinion is highlighting the potential tension clinicians perceive between AI-CDSS recommendations (derived from observational data) and established clinical guidelines or their own clinical experience. To strengthen the implications, please consider elaborating further in the Discussion section on how clinicians might practically navigate such conflicts. Consider discussing the types of training, practice, support, or specific tool features (e.g., clear explanations of data sources and limitations, confidence scores for predictions) that might help clinicians critically appraise and appropriately weigh in different sources of information in their own decision-making process.	Thank you for highlighting this important aspect with regard to this consideration for implementation. We consider that in addition to provision of clear, accessible information and formal training in the use of such tools, reflective practice will play a role in considering how to resolve potential conflicting information in practice. As highlighted in our discussion, this remains an area that would warrant further research.	Discussion. Findings in context, Para 2. Amended/expanded.<br><br>*"Communicating this to users of AICDSS through provision of accurate, accessible and tailored information and training would support development confidence and proficiency to accurately judge different information sources against each other. This could be achieved by e-learning training and through integration AICDSS training in the curriculum for HCPs in training. Where issues arise in applying potentially conflicting information, transparency in what data a recommendation is based on is important, and to the extent possible, how the AI made the recommendation.*

		In a wider perspective, this also highlights the importance of transparency and regulation in AI development and implementation at large. Research in this area is urgently needed."
Practical Facilitation of Shared Decision-Making (SDM): The study provides a thoughtful exploration of the tool's potential role in SDM, noting varied views among clinicians. I recommend enhancing the practical applicability of the findings, by considering a slight expansion on how the OPTIMAL tool (or even similar AI-CDSSs) could be more effectively adapted, tweaked, customized, presented, or integrated into diverse clinical workflows (including remote consultations) to genuinely and practically facilitate SDM. This could include discussing strategies to accommodate the varying levels of information desired by patients and clinician concerns about consultation time	Thank you for your interest in this aspect of the research. The OPTIMAL team have specifically considered how outputs from the tool could be presented to patients, compared to clinicians, and this is covered in a separate manuscript being prepared for publication based on interviews with patients living with multiple long term conditions.<br><br>Addition of the reference for the now published study from OPTIMAL team which explored approaches to managing multiple long term conditions including the information desired	Background. Para 4.<br>Reference added to Cooper et al. with minor amendment to text:<br>*"This study builds on previous research by the OPTIMAL team exploring clinicians' perceptions of managing MLTC and their attitudes towards using AI tools to support shared decision making"*
Reviewer 2		
Abstract – in your design, please specific the type of thematic analysis you used.	As per the longer comment and reply below, we have amended the abstract accordingly.	Abstract:<br>*"Design: Qualitative study using <u>template analysis</u> of semi-structured interviews, based on a case vignette and a mock-up of an AICDSS (the OPTIMAL tool)."*
In your methods, it wasn't clear how the OPTIMAL mock-up used during your interviews was developed. You mention about stakeholder involvement but could be more specific on who was	Thank you for this comment. We have attempted to address it as per below, reflecting the largely pragmatic approach to the mock-up	Data Collection, para 4<br>*Vignettes have previously been used in evaluating clinical applications of AI in MLTC [18]. In parallel, a mock-up of the proposed clinical decision*

involved. I would expect to see a proposed theory of behaviour change or set of guiding principles, based on what is already known from the wider literature. This would then have informed the mock-up concept for your initial intervention. I recommend having a look into the Person-Based Approach to intervention development – you don't have to use this method, but hopefully this shows a way to clearly define how the intervention developed prior to this study.	and vignette creation shared with the wider OPTIMAL project (and references in the shared and published protocol, see in-text ref). We read the article referred to on PBA with interest – certainly PBA would have offered a more comprehensive, implementation-research-informed base for the vignette used and we will bear this is mind for future similar endeavours.	*support tool (Figure 1) was shared onscreen with participants. It included summary medical and prescribing information, measures of the effectiveness of comparator treatments in a group of patients with similar clinical characteristics, and estimated effects on current long- term conditions, and the most likely next conditions. Our vignette was created through an iterative process through discussions between the practicing clinicians (including 4 GPs) in the OPTIMAL team and the PAG. The mock-up was then developed through iterative discussions between the clinicians, and a software engineer and the PAG, based on a literature review of existing shared-decision-making tools including QRISK. Several ways of representing data through visual means were trialled and iterated, and those that most effectively conveyed the outputs, and were accessible to the clinicians and the PAG were used. This was done as part of the wider OPTIMAL project [15] ."*
I recommend reading the following paper (and other similar papers) that also use vignettes to discuss the use of AI clinical tools - Value of using artificial intelligence derived clusters by health and social care need in primary care: A qualitative interview study with patients living with multiple long-term conditions, carers and health care professionals.	Thank you for this interesting recommendation. AI-augmented clustering of MLTC certainly holds potential to improve management of strategies for these patients in primary care. We have referred to this article in the methodology section	Discussion/Preceding scholarship. New paragraph: *"Concerns of lack of human interaction and subsequent personalisation of interventions were reflected in a recent interview study with MLTC patients and their carers (your ref), but likewise, AI was seen as potentially very useful*

https://pmc.ncbi.nlm.nih.gov/articles/PMC12188064/	reg. the usage of a vignette, and in the Discussion (see Right).	*in navigating complex clinical and social needs."*
I think the methods section needs more work. You mention using an inductive thematic analysis but I wonder if this really fits what you have described. I recommend reading the Braun and Clarke article (Toward good practice in thematic analysis: Avoiding common problems and be(com)ing a knowing researcher). There is a section around the difference between topic summary themes and meaning-based interpretive story themes which will be useful. I think your themes as currently presented as topic summary themes, and tie directly into your project aim/questions as your theme names/areas. This is fine (and very useful in pragmatic research) but potentially lends itself more to codebook thematic analysis than inductive thematic analysis e.g. you had a specific question/set idea of the things you were looking for within the data rather than being guided by narratives within the data. I think the methodology could be made clearer and be more detailed to enable replication of analysis approach	Thank you for this useful feedback. The thematic analysis is founded on the concepts introduced in the topic guide, and organised by the primary- and secondary aim. There was an element of inductivity in how the subthemes were derived from the interview data, we agree that the overarching approach to the analysis is what Braun and Clarke refer to as *Codebook Thematic Analysis*.<br>In response to your comment, we have clarified this using a different terminology and now refer to our methodology as Template Analysis (*King, N., & Brooks, J. M. (2018). Thematic analysis in organisational research*). Whilst we appreciate that there is an element of a post-hoc classification through doing that, the approach described by King and Brooks closely mimic what we de facto did i.e. create an early coding template based on aim and topic guide and reiteratively modify it, using a largely deductive approach but allowing for some inductive flexibility, in particular (but not only) with regards to the secondary aim.	Data analysis, para 1 and 2: *"After the interviews had been conducted the data were coded by the researcher who had conducted them. NVivo 14 software was used to manage the data, with verbatim transcripts imported. Data were analysed through template analysis, a form of codebook-led thematic analysis as described by King and Brooks (2018). Template analysis was chosen as it allowed first the development of codes to meet the primary aim of the study to be deductively applied to the data, whilst subsequently allowing for inductive flexibility in exploring emerging themes in the data (in particular supporting the secondary aim as stated in the Aims section above).*<br><br>*An initial coding template with themes was created based on the topic guide and our initial familiarisation with the data. The themes were organised into two overarching groups reflecting the aims: 1) Views on the OPTIMAL tool and its practical implementation in clinical practice; and 2) The interpretation and application of the outputs from an AICDSS in general such as the OPTIMAL tool. "*

"Themes were thereafter compared through consensus meetings and similar themes collapsed into each other." – In this sentence, I think you mean similar codes collapsed and not themes	Good point. See amendments to the right:	Data Analysis, para 3:<br>Clarified:<br>*Two interviews were coded by both researchers (AD and GM) and reviewed for consistency. Codes were compared through consensus meetings as similar codes emerged. Throughout the coding process, themes were then modified to reflect the codes derived from the data, and subthemes were created to describe the findings in detail.*
The themes within the text are not clear – your supplementary Table 3 presents your findings more clearly. I think you could find a way to describe your themes and subthemes using the format of your table	Thank you for pointing this out, and on reflection it could certainly be made clearer. We have slightly reworked the section, mainly the introduction to the Results, to address your points. See amendments to the right:	Results, introduction:<br>The table from Supp 3 has now been integrated into the main text and acts as a support for the introduction of the Results section.<br>We have reworded the introduction as well as the two main headings of the results section to better reflect how they discuss the primary and secondary aim, and we have clarified that the subthemes are discussed in the running text of the respective theme.
Limitations – you reflect on the limitations of geographical area but could expand on this in terms of potential impact on implementation e.g. IT systems vary by practice and by region, complicating the ability to embed integrated, consistent tools.	Thank you for this. Please see alterations to the right.	Discussion/Strengths and Limitations, para 2:<br>*"Whilst our results are likely to be applicable beyond this geographical area, differences in local EHR systems, prescription policies, patient population and clinical pathways means caution is required when extrapolating. However, the proposed OPTIMAL tool as studied is not system specific. In addition, the majority of the interviewees had previously participated in a related,*

		currently unpublished, interview study on the views of AI in MLTC in general. This may have affected their answers in our study"
Limitations – you reflect on the quality of CPRD Aurum coding (which was reflected in your data). You could also reflect on the wider impact of AI tools on health inequalities within your limitations section	Thank you for this comment.<br>Whilst data quality in CRPD Aurum is known to have limitations (although big data models can overcome some of this based on enormous sample sizes), it did not specifically affect our study. These limitations of CPRD Aurum as well as the risks with regards to health equity are as such discussed herein rather as a reason for failed implementation and low clinician trust.<br>Therefore, we have chosen not to discuss this in the limitations section, as we believe it may veer from the scope of the study into a much wider discussion on the clinical suitability of AICDSS in general.	No amendments made, but we just want to highlight this existing section in the Preceding Scholarship section:<br><br>*"Numerous precedents exist where AI- or big-data based CDSSs have been found to produce different accuracy and effectiveness for different demographic groups, potentially widening health inequities [28]."*
It was great to see your PAG mentioned and the input they had. I think you could provide some more specific information to strengthen this section e.g. how many members are in your PAG? Broadly, what lived experiences do they represent?	Thank you for your kind words.<br>We have provided more information as requested, using the phrasing shared within the various OPTIMAL project studies.	Data collection, para 2:<br>*"The interview topic guide (supplement 2) was developed in collaboration with the Patient Advisory Group (PAG) for the OPTIMAL study (reference) and the wider OPTIMAL team. The PAG consisted of nine members who were either living with MLTCs (four or more long-term conditions) or caring for people with MLTCs, with experience of common as well as rare and both physical and*

		mental conditions, and with variation in personal characteristics (age, sex, ethnicity). It provided indicative questions and prompts covering perspectives on shared decision making, the use of non-AI clinical decision support tools, and perspectives on the proposed OPTIMAL tool. The topic guide was piloted on three GPs which led to minor semantic adjustments."

VERSION 2 - REVIEW

Reviewer 2

Name Holt, Sian

Affiliation University of Warwick Warwick Medical School, Division of Health Sciences

Date 14-Oct-2025

COI

Thank you for addressing the comments so thoroughly. I have two more minor comments.

In the data collection section, you have a missing reference on line 29

It might be useful to create a short, summary box or figure highlighting your key messages for a clinicial audience. Something they can see at a glance and take away new learning/ideas for their practice. This isn't a 'must have' but could be useful to consider.

VERSION 2 - AUTHOR RESPONSE

Comment	Amendment
**Reviewer: 2**<br>**Dr. Sian Holt, University of Warwick Medical School**	
In the data collection section, you have a missing reference on line 29	Thank you for pointing this out. However, we on line 29 of Data Collection we cannot find any missing reference, only the existing reference to the preceding OPTIMAL project protocol (technically the qualitative

	component of the OPTIMAL project), reference 15.<br><br>(*Gunathilaka NJ, Gooden TE, Cooper J, et al. Perceptions on artificial intelligence-based decision-making for coexisting multiple long-term health conditions: protocol for a qualitative study with patients and healthcare professionals. BMJ Open. 2024;14:e077156. doi: 10.1136/BMJOPEN-2023-077156*)<br><br>We do of course welcome further clarification and are happy to consider further revisions. No amendments done at this point.
It might be useful to create a short, summary box or figure highlighting your key messages for a clinicial audience. Something they can see at a glance and take away new learning/ideas for their practice. This isn't a 'must have' but could be useful to consider.	Thank you for this feedback.<br>We considered it and made a summary figure. However, within the recommended format of BMJ Open with a brief and conclusive summary section, the abstract, and the existing mandated “Strengths and Limitations” bullet points, we consider this covered within the existing manuscript, and we felt that further similar additions would be duplication.
